# Noninvasive Strategies to Promote Functional Recovery after Stroke

**DOI:** 10.1155/2013/854597

**Published:** 2013-06-24

**Authors:** Alessio Faralli, Matteo Bigoni, Alessandro Mauro, Ferdinando Rossi, Daniela Carulli

**Affiliations:** ^1^Department of Neuroscience, Neuroscience Institute of Turin, University of Turin, Regione Gonzole 10, 10043 Orbassano (Turin), Italy; ^2^Neuroscience Institute Cavalieri-Ottolenghi (NICO), University of Turin, Regione Gonzole 10, 10043 Orbassano (Turin), Italy; ^3^IRCCS Istituto Auxologico Italiano, Corso Goffredo Mameli 197, 28921 Verbania, Italy; ^4^Department of Neuroscience, University of Turin, Via Cherasco 15, 10126 Turin, Italy

## Abstract

Stroke is a common and disabling global health-care problem, which is the third most common cause of death and one of the main causes of acquired adult disability in many countries. Rehabilitation interventions are a major component of patient care. In the last few years, brain stimulation, mirror therapy, action observation, or mental practice with motor imagery has emerged as interesting options as add-on interventions to standard physical therapies. The neural bases for poststroke recovery rely on the concept of plasticity, namely, the ability of central nervous system cells to modify their structure and function in response to external stimuli. In this review, we will discuss recent noninvasive strategies employed to enhance functional recovery in stroke patients and we will provide an overview of neural plastic events associated with rehabilitation in preclinical models of stroke.

## 1. Introduction

Stroke is an acute neurological syndrome caused by disruption of the cerebral blood supply. About 80% of strokes are ischaemic, resulting from an obstruction of blood flow, while about 15% are due to a primary intracerebral hemorrhage. Stroke is one of the leading causes of chronic adult disability and death in western industrialized countries [1]. Neurological deficits reflect the location of the tissue damage and, in particular, the extent of the neuronal loss. Neurons deprived of their normal metabolic substrates cease to function in seconds and show signs of structural damage after only 2 minutes. As energy-dependent processes fail, neurons are unable to maintain their normal transmembrane ionic gradients, resulting in ion and water imbalance that triggers apoptotic and necrotic cell death cascades and, ultimately, leads to focal neurological signs and symptoms. According to the WHO's international classification of function, disability, and health (ICF, WHO 2001), the impairment of brain functions may originate different activity limitations (disability) and participation restriction (handicap).

Motor impairments, including hemiparesis, incoordination, and spasticity, are the most common deficits after stroke. However, functional recovery frequently occurs following stroke, although its extent is highly variable. Some patients with initial severe hemiparesis may eventually achieve full recovery, while others have little or no improvement and remain permanently disabled. There are many reasons for the variable degrees of recovery, including the age of the patient, the location and extent of the lesion, and individual variations in anatomical and functional connections [2].

The neural bases for poststroke recovery rely on the concept of plasticity [3], namely, the ability of central nervous system (CNS) cells to modify their structure and function in response to a variety of external stimuli (experience). The plastic/reparative properties of the brain are determined by the balance between cell-intrinsic mechanisms and extrinsic regulatory molecules, which is regulated by activity-dependent processes and different kinds of interaction with the external world [4, 5]. Molecules in the adult CNS milieu, such as myelin-associated proteins (e.g., Nogo, MAG, and Omgp), factors secreted by astrocytes near the stroke site (e.g., chondroitin sulfate proteoglycans), and repulsive axonal guidance cues (e.g., semaphorins, netrins, and members of the ephrin family), constrain axonal sprouting and hamper the formation of new connections [6]. In preclinical stroke models it has been shown that pharmacological blockade of Nogo, Nogo receptor antagonism, or digestion of chondroitin sulfate proteoglycans by chondroitinase induce axonal sprouting and promote functional recovery [7–9]. Blocking the semaphorin pathway reduces cortical damage after stroke [10]. Other growth inhibitors, such as EphA4 and ephrin-A5, have also recently been identified, which limit functional recovery and are promising targets for repair after stroke [11, 12]. Interestingly, inhibition of ROCK, a downstream target of several growth inhibitors, greatly improves outcome after ischemic stroke [12]. Several studies have also uncovered pharmacological targets that promote a neuronal growth state in the adult CNS. For example, inosine triggers a serine/threonine kinase (Mst3b), enhancing axonal sprouting [13, 14].

The therapeutic potential of replacement strategies in laboratory models of stroke is also under investigation. Transplantation of neural progenitor cells, bone marrow-derived mesenchymal stem cells or human-induced pluripotent stem cells into the ischemically lesioned brain have been proved to be a safe and efficient approach to promote significant functional recovery in experimental animals [15–17]. Nonetheless, the mechanisms underlying the beneficial effects of cell transplantation in the ischemic CNS remain uncertain, and, most importantly, to date there is no clear evidence that donor cells may directly contribute to the structural repair of neuronal circuits.

In addition to pharmacological or replacement therapies, clinical and preclinical studies are currently focusing on noninvasive strategies for post-stroke rehabilitation. Clinical data show that neurologic deficits following stroke can be treated by physical therapy [18]. Motor rehabilitation after hemiparetic stroke typically involves combinatory approaches, including neurofacilitation techniques, task-specific training, and task-oriented training [19, 20]. Furthermore, stroke units, in which patients have access to daily skill training therapies in highly stimulating environments, such as during physical, occupational, or language therapy, result in decreased deficits, increased performance on self-care tasks, lower 1-year mortality, and lower probability to be in a nursing home at followup [21]. Finally, in recent years brain stimulation, mirror therapy, action observation, or mental practice with motor imagery is emerging as interesting options as add-on interventions to standard physical therapies [22]. 

Here, we will provide an overview of recent noninvasive strategies employed to enhance functional recovery in patients after stroke and discuss the current knowledge of rehabilitative strategies and the associated neural plastic events in preclinical models of stroke. 

## 2. Novel Noninvasive Strategies for Patients Rehabilitation

Stroke rehabilitation aims to guarantee that stroke survivors reach the maximum physical, functional, and psychosocial recovery possible within the limits of their impairment. In order to help stroke patients to fully participate in life, the final goal of rehabilitation should be to maximize performance of activities of daily living and independence. Through learning-dependent processes, rehabilitation facilitates and shapes the recovery that would occur spontaneously. Recovery of stroke patients is extremely heterogeneous and determined by a combination of processes including functional restoring of damaged nervous tissue, relearning of lost skills through reorganization of spared pathways (plasticity), adaptation, and compensation for deficits. Compensation reflects the use of alternate behavioral strategies in order to solve a specific task. Most recovery of specific neurological focal deficits occurs during the first 3 to 6 months after stroke, but it is largely accepted that improvements can continue for years after stroke [23].

General principles of stroke rehabilitation include the start of intensive rehabilitation programs carried out in a stroke unit within the first few days after stroke [24, 25]. Evidence demonstrates that comprehensive intensive rehabilitation, as well as the presence of a structured multidisciplinary team, may be more effective than less intense programs [26]. In agreement with the learning nature of the rehabilitative process, involvement, engagement, and motivation of patients, families and caregivers are crucial to obtain good outcome.

Most recent neurorehabilitative approaches are based on a task-oriented model of motor learning, whose main feature is an intensive training with specific tasks in an environmental context (task-specific and context-specific trainings; [27–30]). In this context, a number of new rehabilitative techniques potentially capable of stimulating cerebral plasticity have been proposed and tested in the last years. Among these techniques, large interest is devoted to treatment approaches aimed to improve motor functions, including constraint-induced movement therapy, mental practice, mirror therapy, virtual reality, robotics, and brain stimulation techniques.

Constraint-Induced Movement Therapy (CIMT) involves the restriction of usage of the unaffected limb, forcing the use of the paretic one, and aiming to contrast the maladaptive “learned nonuse” of the paretic limb (the subject learns to ignore the damaged limb because of its lack of functionality and learns to use exclusively the healthy limb). A number of studies including randomized controlled trials and a Cochrane review have shown that CIMT is effective in improving motor performance in human patients after stroke [31, 32] with a large effect size and robust effects especially on arm function [33]. In particular, the ECXITE trial [31] demonstrated that daily intensive CIMT training for upper limb paresis was superior to the control treatment 3 to 9 months after stroke, and that a modest improvement in motor function persisted in the CIMT group after 2 years. Important limitations to the routine use of CIMT training derive from the fact that it is labor intensive and suitable only for patients with some conservation of motor functions (in particular wrist and finger), thus its use is recommended only for selected patients. 

Mental practice with motor imagery is considered a promising additional treatment to improve motor functions of severely affected upper limb [33], although its clinical effectiveness is not yet clearly proven. This approach grounds on the statement that imaging a movement requires activation of brain circuits involved in the preparation and execution of the same movement and consists in a repetitive cognitive training during which the patient imagines performing a task or body movement without actually physically performing it. It has been demonstrated that mental practice may modulate cerebral perfusion and neural activity in brain regions similar to those activated during actual movements [34, 35]. Following few systematic reviews [36, 37] suggesting that mental practice may be beneficial for post-stroke disabilities in addition to conventional treatments, a recent Cochrane review [38] concluded that there is only limited evidence that mental practice may increase the effectiveness of usual physiotherapy and occupational therapy. 

Another approach based on multisensory stimulation is represented by the mirror therapy. In this technique a mirror is placed at 90° in the patient midsagittal plane, so that the paretic limb is hidden behind the mirror and the patient watches the image on the mirror of the unaffected arm as if it was the affected arm. In a certain sense, the patient receives the impression that the affected limb is functioning. It has been demonstrated [39] that viewing the image of one's moving hand reflected by the mirror increases the excitability of neurons in the ipsilateral primary motor cortex more than directly viewing the inactive hand. Mirror therapy effects (as well as those related to mental practice) may be related to the activity of the so-called mirror neurons, which discharge both following performance of motor acts and simply observing the same action done by another individual [40, 41]. In fact, by means of fMRI it has been demonstrated [42] that prolonged and repetitive observation of an action may enhance the activity in the ventral premotor cortex, the supplementary motor area, and the superior temporal gyrus. A recent systematic review [43] including 14 studies and a total of 567 patients treated with mirror therapy concluded that, when compared to other rehabilitative approaches, this treatment has a significant effect on motor function even though this result is strongly influenced by the type of intervention used as control. Thus, it remains unclear if mirror therapy should replace other treatments for motor rehabilitation after stroke, while its role as additional intervention is confirmed. Moreover, mirror therapy improves activities of daily living, but this statement is limited by the small number of studies (four) examining this effect.

Virtual reality technologies represent a relatively new approach for rehabilitation. The virtual reality idea is based on the possibility that a computer can generate a three-dimensional graphical environment from numerical data [44], so that, by using visual, aural, or haptic devices, the operator can experience the environment as if it were a part of the world. A key feature of all virtual reality applications is interaction: virtual environments are created to allow the user to interact also with virtual objects within the environment. In some systems, the interaction may be achieved via a mouse or a joystick button, while in others, a representation of the user's hand may be generated within the environment with movement of the virtual hand reflecting the user's hand, thus allowing a more natural interaction with objects. Therefore, virtual reality represents a unique instrument to achieve several requirements for effective rehabilitation, such as repetitive practice, feedback about performance, and motivation to endure practice [45, 46]. Specifically, by using virtual reality it is possible to drive and control exercises for patient rehabilitation within a functional, purposeful, and motivating context [45]. Moreover virtual reality technologies play a pivotal role in the construction of telerehabilitation systems.

Different virtual reality approaches have been used, in particular, for upper limb motor rehabilitation. A Cochrane review published two years ago [47], analyzing 19 randomised and quasi-randomised trials of virtual reality that involved 565 participants, concluded that there is a limited evidence that virtual reality and interactive video games may be beneficial in improving arm function and activity of daily living function when compared with the conventional treatments. Another, contemporary meta-analysis [48], including 12 studies (5 randomized controlled trials and 7 observational studies) for a total of 195 patients, showed that in the large majority (11 over 12) of these studies virtual reality added a significant benefit on arm motor recovery after stroke. However, to gain convincing evidence of virtual reality effectiveness in poststroke rehabilitation, further research is needed based on good randomized controlled trials.

In the last years a growing interest has been addressed to robot-assisted rehabilitative treatments after stroke. In theory, robotic devices may help administer an intense repetitive training to facilitate recovery. Several studies have demonstrated a significant result in motor recovery of the upper limb of patients who trained with robotic devices but no significant effect on functional ability [49]. However, the conclusion of a randomized controlled trial (UL-Robot [50]) and a Cochrane meta-analysis [51] limited the significance of these results. In the UL-Robot trial two groups of patients receiving 36 therapy sessions over 12 weeks of robot-assisted therapy or intensive conventional physical therapy, respectively, were compared with patients receiving usual (not intensive) care. The study failed to demonstrate a superiority of the intensive robot therapy when compared to intensive conventional physical therapy, but both techniques were superior to usual care, suggesting that intensity of training may be a crucial factor for motor recovery. The Cochrane review [51], including 19 trials and 666 patients, concluded that electromechanical and robot-assisted arm training after stroke may improve generic activities of daily living as well as paretic arm function, but not arm muscle strength. 

A phase III randomized and controlled trial (LEAPS-[52]), designed to test the efficacy of a popular technique that utilizes partial body-weight support with treadmill training, was concluded in 2011. The LEAPS trial included 408 patients randomly assigned to three groups: two groups were subjected to a locomotor training with treadmill and body-weight support (one group initiating treatment 2 months after stroke and the second 6 months after stroke), the third group received a home exercise program. The results were someway surprising: no significant difference was found between the three groups concerning the improvement in walk speed, motor recovery, balance, functional status and quality of life. Thus, locomotor training with body-weight support and treadmill cannot be considered superior to a structured, progressive, and intensive at home treatment. Also, in this trial all intensive interventions were more effective when compared to non-intensive and structured care.

A promising robotic interface has been recently developed by Courtine's group to evaluate, enable, and train pattern generation and balance during walking in rats. The devise continuously and independently assists or perturbs propulsion and balance along four degrees of freedom, while rats are progressing overground within a large workspace. In a model of stroke, this robotic interface improves equilibrium maintenance, thereby contributing to skilled locomotion [53].

The use of noninvasive techniques of brain stimulation to stimulate adaptive plasticity is very appealing, and the results obtained are exciting. Two main techniques are available to obtain both cortical enhancement and inhibition: repetitive transcranial magnetic stimulation (rTMS) and transcranial direct current stimulation (tDCS). rTMS, using a coil placed on the scalp, generates a focal magnetic field, which induces (transiently, focally, and reversibly) an electric current in the underlying cortex. Low frequency stimulation (in the range of 1 Hz) reduces cortical excitability, while higher stimulation frequencies increase the cortical excitability. In tDCS, weak direct currents are delivered to the cortex through two electrodes that polarize the underlying tissue. Electrode position is crucial to modulate the distribution and direction of the current flow: anodal stimulation has an excitatory effect by cortical neuron depolarization, while cathodal tDCS hyperpolarizes neurons by decreasing cortical excitability. In general, two different approaches can be described using noninvasive brain stimulation: one addressed to increase excitability of ipsilateral damaged hemisphere (e.g., by stimulating primary motor cortex), and the other one directed to reduce the activity of intact surrounding or contralateral area that can produce intra- or interhemispheric inhibition. 

The purpose of these applications is to restore the unbalance between intact and lesioned hemisphere according to the interhemispheric competition model [54]. Moreover, Bestmann and coworkers suggested an unexpected role of the contralesional dorsal premotor cortex, with an elegant demonstration by means of rTMS which showed the supporting activity of contralesional dorsal premotor cortex to ipsilesional sensorimotor regions in particular for greater clinical and neurophysiological impaired patients [55]. The application of these approaches have produced very promising results, in both acute and chronic stroke patients, recently reviewed by Corti et al. [56]. That review suggests that rTMS applied to the affected hemisphere is safe and could be considered effective for modulating brain function and contributing to motor recovery after stroke. However, the authors stressed the need of double-blinded, sham-controlled Phase II and Phase III clinical trials involving larger sample sizes to validate this treatment. In a meta-analysis of 18 randomized controlled trials dedicated to the effects of rTMS on upper limb motor impairment, Hsu et al. [57] found a significant effect size (0.55–95% CI, 0.37–0.72) for motor outcome function, with more clear effects for subcortical stroke and low-frequency rTMS applied to the unaffected hemisphere. Talelli et al. questioned about the real duration and anticipated size of the treatment effects in chronic stroke patients. In such patients they showed with a small semirandomized clinical trial that rTMS application does not augment the gains from a late rehabilitation program [58]. The need for randomized controlled trial is even more evident to validate efficacy of tDCS, considering that its use in stroke patients is quite new [59, 60]. Recently, Khedr et al. provide an interesting evidence that both anodal and cathodal tDCS are superior to sham stimulation in enhancing the effect of rehabilitation training to improve motor recovery after subacute stroke in a pilot randomized controlled trial [61]. However, it must be stressed that our knowledge about mechanisms underlying brain stimulation are largely incomplete. Thus, different paradigms of brain stimulation will likely appear in the next future. 

## 3. Noninvasive Therapies in Animal Models of Stroke

### 3.1. Enriched Environment

**Figure 1:**
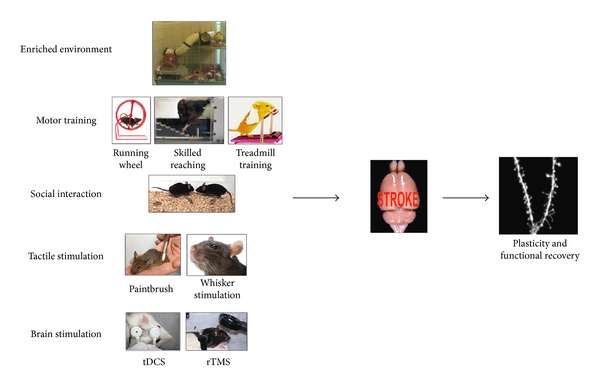
Figure 1 summarizes some of the most used noninvasive strategies to promote neural plasticity and functional recovery in experimental models of stroke.

Rehabilitative conditions in stroke units, such as physical therapy and various kinds of stimulating activities, can be partially mimicked in animal studies by housing the animals in an enriched environment (EE). EE is a widely employed paradigm to study the influence of external stimuli on brain plasticity in animal models both in physiological conditions and after damage [62]. Environmental enrichment refers to housing conditions that facilitate enhanced sensory, social, cognitive stimulation, and motor activity. Home cages used for enrichment are larger than standard cages to allow room for several objects, which generally vary in composition, shape, size, texture, smell, and colour. Enrichment may also involve access to running wheels for enhanced voluntary exercise (Figure 1). Key aspects appear to be the provision of environmental complexity, with objects that offer a range of opportunities for visual, somatosensory and olfactory stimulation, and environmental novelty, obtained by changing the objects and their position in the cage, which might provide additional cognitive stimulation. Increased complexity and novelty also lead to greater levels of physical activity. Social interactions are also favored by housing rather large groups of animals of both sexes together (see for review [63]). Several studies show that in experimental models of stroke, EE strongly promotes recovery of motor functions, such as skilled limb function [64–68] and gait [69]. Compensatory mechanisms have been shown to substantially contribute to functional improvement after stroke [70, 71]. Compensation reflects the use of alternate behavioral strategies in order to solve a specific task [70, 72]. To what extent EE enhances functional outcome after stroke due to compensation for lost functions rather than their restoration is not entirely clear. Witte and coworkers addressed this question by focusing on the time course of functional recovery versus motor compensation in skilled forelimb movements after cerebral ischemia in rats. The skilled reaching task allows the distinction between recovery and compensation by quantitative (reaching success) and qualitative (movement pattern) analysis. It has been shown that EE facilitates effective compensation in skilled reaching, while it does not promote restitution of function. Namely, rotating movements of the forelimb during reaching are permanently impaired and require functional compensation through intensified use of the upper body [68].

Interestingly, in one of the first studies on the effects of EE on stroke animals, Ohlsson and Johansson [64] addressed whether preoperative and postoperative environments can differently influence functional outcome after focal brain ischemia. Rats were subjected to ligation of the right middle cerebral artery (MCA) then transferred from a non enriched to an enriched environment or reared in an EE already before the operation. Rats kept in an EE before and after the MCA ligation improved sooner and to a slightly higher degree than those placed in the EE only after the ischemia. The beneficial effects of EE in the animals enriched before MCA ligation suggest that complex experiences during healthy conditions may provide a “brain reserve” against late brain damage, according to previous findings [73–78]. Among the EE-induced changes in physiological conditions, the development of new synapses [79, 80] and dendritic spines [81–83] has been demonstrated. In addition, there is evidence that exposure to EE reduces the expression of growth-inhibitory molecules in the intact CNS tissue [84, 85]. Therefore, it is conceivable that reduced inhibitory mechanisms together with a “reserve” of synapses in enriched animals may provide neuroprotection and facilitate functional compensation after stroke.

### 3.2. Motor Training

A bulk of evidence highlights the functional benefits induced by motor training after focal ischemic injury in humans. A useful method of training for chronic and acute individuals after a stroke is treadmill training [86] (Figure 1). When applied to ischemic rats starting 24 h after ischemia, it leads to a significant reduction of infarct volume and improves neurological function [87]. Interestingly, functional recovery after stroke (such as forelimb foot placing, parallel bar crossing, and rope or ladder climbing) can be further improved by complex motor training (which can be obtained by using rotarod) rather than simple repetitive exercise, such as treadmill training [88]. This suggests that repeated complex movements involving motor balance and coordination are more effective for functional recovery after stroke than either simple activity or inactivity.

In line with this view, specific behavioral experience, such as skilled-reaching training (Figure 1), after focal experimental infarct, provides substantial behavioral recovery of skilled hand function in monkeys [89]. In experimental animals, skilled reaching training consists of daily practice of the impaired forelimb to retrieve food pellets. This kind of rehabilitation provides positive reinforcement (i.e., food reward) associated with use of the impaired limb, thereby encouraging animals to practice “spared” motor function or promoting development of compensatory motor strategies, resulting in lessened functional deficiency.

Interestingly, by combining both enriched living conditions and daily skilled-reaching training, Biernaskie and Corbett [66] obtained dramatic long-term improvement both in skilled use of the impaired forelimb and digits and in limb placement in stroke rats. These findings reinforced the idea that skilled learning therapy coupled with enriched surroundings may facilitate neurologic recovery in humans. It should be noted, however, that the effect of forced exercise on functional recovery after stroke is controversial. Forced exercise, such as treadmill running or constraint-induced movement therapy, has been shown to enhance the functional recovery of motor skills after experimental ischemic stroke [90, 91]. Other studies, however, demonstrate that treadmill running produced negative physiological adaptations induced by stress [92], and a constraint-induced movement study did not show improved functional outcome after brain ischemia [93]. 

### 3.3. Social Stimuli

Patients with high levels of social support or large social networks exhibit more rapid and extensive functional recovery after stroke than socially isolated individuals [94, 95]. The importance of social influences on stroke outcome have been also highlighted in experimental animals by Johansson and Ohlsson [96] (Figure 1). These authors assessed the relative importance of postoperation physical activity and social interaction for functional outcome. Rats were housed together in a large cage with no equipment or housed individually in cages with free access to a running wheel and compared to rats kept in an EE. Interestingly, rats housed together in a large cage with no activity-stimulating facilities improve more than rats housed in individual cages with access to a running wheel. However, rats housed in an EE improve significantly more than the other two groups, suggesting that, although increased physical or social activity alone might result in some of the beneficial effects observed with enrichment, they do not fully account for the broader behavioural improvements observed following exposure to complex stimuli.

To study social influences on experimental stroke outcome, DeVries' group addressed the effects of social isolation versus pair housing on stroke-induced infarct size and functional recovery in mice. They observed that pair housing decreased infarct size and improved functional outcome of stroke mice when compared to socially isolated mice [97]. Social interaction influences locomotor activity [98] and introduces auditory, olfactory, and visual stimuli, which in turn may influence pathophysiological mechanisms and recovery. Further, the same authors asked whether one aspect of social interaction, namely, physical contact, may mediate the effects of social interaction [99]. To control for the element of physical contact during pair housing, the experiment included the use of standard cages fitted with a grid partition that allowed the experimental mouse to see, hear, and smell its partner but not engage in physical contact. Interestingly, only paired animals that were in unobstructed physical contact showed smaller infarct volumes and exhibited recovery of locomotor activity following MCA occlusion, indicating that physical contact during social interactions influences stroke outcome. Further clinical research is, therefore, needed to determine the influence of physical contact on patient recovery.

### 3.4. Tactile Stimulation

Another potential noninvasive treatment that might have a significant impact upon recovery of skilled motor behaviors after stroke is tactile stimulation (Figure 1). When stroke rats are given tactile stimulation, which involves petting animals individually with a baby hairbrush or a paintbrush, they show dramatic improvement in the single pellet reaching task relative to untreated lesioned animals [100]. These data suggest that massage therapy might be beneficial in resolving motor deficits in human stroke patients.

Interestingly, intermittent single whisker stimulation, if initiated within 2 h of permanent MCA occlusion in the rat, induces complete protection from ischemic stroke by 24 h after injury, preventing the expected damage and deficits. Namely, animals that receive early stimulation treatment show no sign of infarct. An initial absent or severely disrupted whisker functional representation is followed by gradual recovery to baseline responses over the treatment period. Evoked subthreshold activity and spiking and blood flow levels, which are severely decreased immediately after occlusion, return gradually to preocclusion levels. Blood flow data suggest that the protection induced by early stimulation is due to reorganized blood flow via collateral vessels (interarterial connections). In contrast, animals that do not receive treatment until 3 h post-MCA occlusion show compromised function and large infarcts [101, 102]. These studies raise hope for the development of stimulation-based strategies to mitigate stroke pathology in humans.

### 3.5. Noninvasive Brain Stimulation Techniques

#### 3.5.1. tDCS

Recent studies employed animal models to investigate the positive effects of tDCS and define the optimal time window of its application after stroke (Figure 1). Both early (1 day after ischemia) and late (1 week after ischemia) anodal tDCS treatments exert beneficial effects on cognition, behavioral function (i.e., improved Barnes maze performance and motor behavioral index scores), and neural plasticity, without exacerbating ischemic volume and metabolic alteration [103]. However, only the rats receiving late tDCS treatment showed improvement in the beam balance test [103]. Accordingly, in the study by Jiang et al. [104] anodal and cathodal tDCS applications from day 1 to day 3 after cerebral infarction do not improve the beam walking test scores of rats on day 3, but significant amelioration of motor function is observed if the animals receive continuous application of tDCS till day 7 or 14. These findings suggest that late application of tDCS may result in stronger motor function improvement than earlier intervention after stroke. Accordingly, one study, in which anodal tDCS was applied during five daily sessions to the ipsilesional primary motor cortex in acute stroke patients starting on the 2nd day, did not reveal any significant difference in motor function between the tDCS and sham groups, indicating that tDCS application from day 2 to day 5 after stroke does not promote functional recovery [105]. LTP and LTD may be candidates processes to explain the cellular correlates for tDCS-induced effects [106, 107].

#### 3.5.2. rTMS

Despite the observed beneficial effects in humans (see for review [108]), the cellular/molecular mechanisms underlying rTMS action are far from clear. It is likely that rTMS induces LTP or LTD, which, in turn, produce enduring changes on neocortical excitability and synaptic connections [109–111]. In humans, an increase in motor-evoked potential amplitude [110, 112], regional cerebral blood flow, glucose metabolism [113], and EEG response amplitude [109] has been reported. Studies in animal models (Figure 1) have shown that rTMS effects depend on changes in NMDA receptor activity [114]. Interestingly, Wang et al. [115] provided the first evidence that rTMS induces changes in BDNF-TrkB signaling in the rat brain, which are reflected in lymphocytes. Transcription of glial fibrillary acidic protein (GFAP) is increased in astrocytes of the mouse dentate gyrus (the magnitude of this response depends on the number of stimulus trains), suggesting that rTMS induces the first stage of a reactive response that is similar to what occurs following nervous tissue injury [116]. However, the consequences of rTMS on experimental animals after stroke have been poorly investigated. Zhang et al. [117] report a significant recovery of neurological severity score in stroke rats treated with TMS, which is accompanied by increased expression of c-Fos and BDNF in the cerebral cortex surrounding the infarction areas.

## 4. Is There a Critical Period for Successful Rehabilitation?

After clinical stroke, the initiation of physical rehabilitation programs varies from days to several weeks after the insult. Determining whether there is a period during which the poststroke brain is most sensitive to physical rehabilitation is essential to maximize the functional gains from such therapy. Biernaskie et al. [118] hypothesized that implementing rehabilitative treatment early after the stroke would enhance functional outcome. To characterize a potential “critical period” for successful rehabilitation after stroke, animals received enriched rehabilitative training at 5 d, 14 d, or 30 d after MCA occlusion. Early initiation of enriched rehabilitation (5 d after stroke) provides enhanced functional outcome relative to ischemic animals receiving delayed rehabilitation, suggesting that the poststroke brain is in a state of heightened sensitivity to behavioral experience. In line with those findings, Barbay et al. [119] demonstrate a time-dependent, rehabilitation-induced map reorganization after ischemic injury in primates. Similarly, early treadmill training (started 24 h post-MCA occlusion) was found to have significant effects in reducing brain infarct volume and in improving neurologic function, when compared with late training (started 1 week post-MCA occlusion, [87]). Nevertheless, some evidences suggest that early training after focal brain ischemia in rats exacerbates brain damage and worsens the general outcome after excessive use of the impaired limb. Namely, when the intact forelimb is constrained immediately after the surgical procedure, thus forcing the animal to overuse the impaired forelimb for postural support and movements, functional improvement is reduced [120, 121]. The intensity of training may contribute to early exclusive use-dependent exaggeration of injury. For example, in the study by Yang et al. [87], the intensity of treadmill training for 30 min/day seems to be mild compared to forced use by casting procedures. Excessive sensorimotor activation too early after the insult may exacerbate injury through a use dependent, NMDA-mediated process, possibly stimulating an excitotoxic cascade [122]. This process may dissipate over days, explaining why rehabilitative experience beginning 3–5 d after insult does not worsen injury size or behavioral outcome [89, 123]. In addition, during the first week after injury, the tissue surrounding the infarct is reported to show decreased phasic inhibition and thus become hyperexcitable [124]. However, Carmicheal's group show that while phasic GABA signaling is reduced in the first weeks after stroke, tonic GABA signaling is potentiated in peri-infarct motor neurons. Behavioral and electrophysiological studies in mice suggest that the overall effect in terms of motor cortex circuitry is a diminished neuronal excitability, which when reversed leads to recovery. Therefore, the precise signaling systems in brain excitability that are deleterious in the early phases, become beneficial in later phases of recovery (see for a comprehensive review on brain excitability in stroke [125]). Rehabilitation may act by affecting this delicate balance between hypo- and hyperexcitability of neuronal circuits in peri-infarct cortex.

Interestingly, immediate exposure to EE improves functional outcome, despite exacerbation of ischemic injury [67, 126], perhaps as a consequence of removal of functionally abnormal neurons. Nonetheless, early EE combined with training enhances recovery when compared with conditions in which rehabilitation is started later and is not accompanied by any exacerbation of injury [118]. In addition, a “window of opportunity” extends also to neurovascular changes, which can facilitate full protection [101].

In summary, the efficacy of rehabilitative therapy after stroke is influenced by the time of its initiation, with mild intensity physical training provided early after brain injury being beneficial for functional improvement. Delaying the beginning of rehabilitation may instead reduce the efficacy of treatment and, as a consequence, more intense or longer duration therapies are required to achieve the same functional gains. 

## 5. Cellular and Molecular Correlates of Rehabilitation-Induced Plasticity

### 5.1. Neuritic Plasticity, Reorganization of Connectivity, and Circuit Rewiring

Much of the recovery after stroke is likely due to brain plasticity, with some areas of the brain taking over the functions previously performed by the damaged regions. Proposed mechanisms include: (i) redundancy of brain circuitry with alternative pathways taking over when another one has been damaged; (ii) unmasking of previously existing but functionally inactive networks; (iii) sprouting of fibers from surviving neurons with formation of new synapses [127, 128]. The mechanisms involved likely depend on the extent of injury. When damage to a functional system is partial, within-system recovery is possible, whereas after complete destruction, substitution by a functionally related system may be the only alternative [129].

In stroke patients, improved arm and hand movement and clinical scores have been found in correlation with an enlargement of the hand region in the ipsilesional cortex [130–135]. However, the exact mechanisms behind these changes remain elusive. Activity changes in specific cortical areas may result from a reduction in inhibition from horizontal or callosal connections [136]. Alternatively, new connections may form due to lesion-induced sprouting at the cortical or subcortical level [137, 138]. Reorganization of neuronal connectivity around the lesion site and also in the undamaged contralateral cortex has been detected [139–141]. Interestingly, following an ischemic subtotal lesion of the rat forelimb motor cortex, spontaneous recovery of forelimb function is correlated with hindlimb corticospinal neurons forming new connections with cervical, forelimb-related, spinal cord neurons [142]. 

Rewiring of connections after stroke is further enhanced by rehabilitation. For example, while an ischemic lesion confined to a small portion of the representation of one hand results in a further loss of hand territory in the adjacent, undamaged cortex, early rehabilitative training prevents the loss of hand territory adjacent to the infarct. In some instances, the hand representation expands into regions formerly occupied by representations of the elbow and shoulder. Functional reorganization in the undamaged motor cortex is accompanied by behavioral recovery of skilled hand function [89]. Moreover, stroke rats housed in an EE or receiving tactile stimulation [100] have significantly increased dendritic branching and spine density on pyramidal cortical neurons than control stroke rats, suggestive of increased sprouting of intracortical connections in the enriched/stimulated group [143]. Indeed, EE can influence a number of factors, such as functional enforcement of existing neuronal circuits, sprouting, formation of new connections, and angiogenesis [63, 144]. EE may also modulate ischemia-induced glutamate excitotoxicity, thus leading to attenuated oxidative damage and neurodegeneration [145]. One candidate mechanism underlying the beneficial effects of EE on functional recovery after stroke involves upregulation of neurotrophic factors [146, 147], which may stimulate neuritic remodeling and synaptogenesis. 

Cortical neurons that sprout a new connection after stroke activate a neuronal growth program that consists of transcription factors, cell adhesion, axonal guidance, and cytoskeletal modifying molecules [148]. It is known that EE modulates the expression of several genes in the infarcted cortex [149]. Namely, postischemic EE or social interaction modulate the expression of substances associated with neuronal plasticity, such as nerve growth factor-induced gene A (NGFI-A) and NGFI-B. NGFI-A (also known as Egr1, krox24, zif/268, and TIS8), a transcription factor belonging to the early growth response family [150], is associated with stabilisation of LTP and learning [151, 152]. NGFI-A target genes are synapsin-I and -II, which are involved in synaptic vesicle trafficking and release as well as synaptogenesis [153–155]. Synapsin-I and –II are increased in the ipsilateral cortex of stroke rats following skilled training [156]. In addition, NGFI-A is a master switch for the initiation of inflammatory gene expression under ischemic stress [157]. NGFI-B (also known as Nur77, N10, TIS1, or TR3), a member of the steroid/thyroid receptor family without any known ligand [158], has also been associated with LTP [159]. At one month following MCA occlusion the mRNA expression of NGFI-A and NGFI-B is increased after EE in the cerebral cortex and the hippocampus [160]. However, other reports show a decreased expression of NGFI-A in both cortices of EE rats [161–163], likely reflecting the suppression of postischemic inflammation in the brain. Differences in the intensity and the duration of exercise administered to the rats may account for the different results obtained. 

### 5.2. Compensatory Neurogenesis

Postlesional plasticity in the adult brain is not restricted to structural modifications at the level of axons, dendrites, and synapses but also comprises the generation, differentiation, and maturation of new neurons in circumscribed brain regions (reviewed by [164]). Numerous studies utilizing different experimental models have shown that an ischemic CNS lesion leads to a substantial increase in proliferation of neural stem cells and subsequently increased generation of new neurons in the subgranular zone of the dentate gyrus and in the subventricular zone (SVZ) (see for review [143]). Dentate neurogenesis is stimulated by focal ischemic infarcts even when the site of the injury is located in remote cortical brain areas [165, 166]. Newborn neurons in the SVZ are recruited to infarcted areas and may start to express region-specific mature neuronal markers [167–172]. However, newborn cells expressing mature and region-appropriate neuronal markers have only been observed in the ischemic striatum but not in the cerebral cortex, with low fractions of newly generated cells surviving into maturity [167, 168, 173]. Possible reasons for the reduced incidence of neuronal replacement in the ischemic striatum and cortex could be low cell survival or hampered neuronal phenotypic maturation due to detrimental factors in the perilesional environment, lack of neurotrophic support and of necessary developmental cues. Notably, ablation of doublecortin-positive neuronal precursors from the rostral SVZ and dentate gyrus abolishes neurogenesis and associated neuronal migration induced by focal cerebral ischemia. This results in increased infarct size and worsened neurologic deficits, indicating that neurogenesis contributes to neuroprotection and short-term functional outcome after experimental stroke in mice [174]. Those beneficial effects may depend on the release of chemical mediators (e.g., growth factors) by immature neurons [175].

Studies on the effects of EE and exercise on the adult germinal niches in intact animals have shown that both these paradigms lead to increased neurogenesis in the hippocampus and the SVZ [63]. However, environmental and physical activities affect the lesioned brain differently. For example, postischemic EE enhances cell proliferation in the SVZ, with stronger effects in the chronic poststroke phase [171, 176], while wheel-running exercise after neocortical infarction attenuates the early poststroke activation of the SVZ germinal niche [176]. Interestingly, no effect of EE or exercise on hippocampal progenitor cell proliferation is reported after transient global ischemia in rats [177], suggesting that common pathways of regulation by lesion and environmental interventions may exist [178–180]. In contrast, specific rehabilitative training of the impaired forelimb (skilled reaching training) is able to increase dentate neurogenesis relative to nontrained stroke rats, although at lower levels when compared with sham-operated animals. Moreover, increased levels of newborn granule cells generated in the dentate gyrus correlate with better functional outcomes [181, 182]. 

Interestingly, postischemic EE combined with spatial learning (which simulates occupational therapy in human rehabilitation and activate hippocampus and prefrontal cortex) restores the perturbed dentate gyrus neuroblast production resulting from focal ischemic insult and increases neuroprotection in the ischemic penumbra [183].

### 5.3. The Contribution of Glial Cells to Postlesion Plasticity and Repair

The lack or inadequacy of endogenous neuronal replacement after brain lesions encouraged investigations on the role of glial cells in poststroke recovery process. Increasing evidence indicate that glial cells crucially contribute to the degenerative and regenerative processes following ischemic brain lesions [184, 185]. Also, some of the beneficial effects of EE on the postischemic brain might be mediated by a dynamic modulation of different glial populations.

It is well known that astrocytes are essential for optimal neuronal function and take an active part in synaptic generation and plasticity as well as in maintenance of neuronal and synaptic homeostasis [186–188]. Recently, it has been revealed that astroglia may represent neural stem cells in the adult brain and may also direct neuronal differentiation of adult neural stem cells [189–191]. 

After brain insults like stroke, astrocytes play a multifaceted role [184]. They immediately proliferate in response to the lesion, increase their expression of GFAP, and contribute to the formation of the glial scar [192, 193]. Reactive astroglia might provide a protective environment in the perilesional zone by shielding neurons from oxidative stress [194, 195] or producing antiapoptotic and trophic factors. Accordingly, they might promote neuronal survival, synaptic remodelling, and neurite outgrowth [184, 196–199]. Postischemic EE or daily training of the impaired forelimb enhances astrogliosis in the perilesional area [171, 176, 193]. Reactive astroglia, although representing an impediment for axon growth, may fulfill important protective and reparative functions after ischemic injuries and rehabilitation [199–203]. 

Immediately after the ischemic insult, resting microglia change their morphology from a ramified to an activated hyperramified phenotype and express the CD68 antigen [204]. The activated microglia migrate towards the lesion, remove the necrotic tissue by phagocytosis, and thereby become macrophages [205, 206]. Some macrophages derive from monocytes that cross the blood-brain barrier after the ischemic lesion [207, 208]. Besides the degradation of necrotic cells, activated microglia and macrophages release growth factors and scavenge-free radicals [209, 210]. However, activated microglia could also harm the injured brain with the synthesis of potentially toxic substances like nitric oxide and reactive oxygen radicals or the release of glutamate and proinflammatory cytokines [209, 211–216]. Indeed, recent studies show that suppression of activated microglia and macrophages significantly improve functional recovery after focal ischemic infarcts [217, 218]. In stroke animals exposed to EE or training a reduction of proliferating microglia and macrophages is observed, which may favor the better functional outcome observed [193].

Finally, proliferation and survival of immature and mature oligodendrocytes are only slightly influenced by EE. It has been shown that EE increases the number of NG2-positive glia, in intact ipsi-and contralateral cortical regions remote from the infarct [219]. NG2-positive cells possess some characteristics of multipotent progenitor cells, may support neuronal function, and can turn into myelin-forming oligodendrocytes [220–223]. However, the role of this cell population in the injured brain is still obscure.

## 6. Conclusions

Novel noninvasive interventions for stroke patients, such as mental practice, mirror therapy, virtual reality, robotics, and brain stimulation techniques, are emerging as potentially efficient strategies to promote functional recovery, but in most cases only when provided in combination with physical rehabilitation [43, 224]. The expansion of rehabilitative programs with a wide range of possible interventions is more likely the key to obtain optimal results, by stimulating different reparative and adaptive brain processes. Particularly, the use of noninvasive techniques of brain stimulation to promote adaptive plasticity, such as tDCS and rTMS, is very appealing, and the results obtained in preclinical and clinical models of stroke are exciting. In this context, however, randomized controlled trials are needed to validate the efficacy of these techniques. Moreover, a deeper understanding of the underlying mechanisms is necessary. This knowledge may allow the identification of biological markers suitable to monitor plastic processes in human patients undergoing specific rehabilitative programs, predict the outcome of the treatments, and optimise existing procedures. In conclusion, in the last few years there has been an enormous progress in the field of rehabilitative trials after stroke, for example, in terms of standardized interventions and tools for assessment of function and patient selection (e.g., recruitment of homogeneous groups of patients). Crucial issues, however, remain to be addressed in future studies, including the sample wideness, repeatability of the results, and effective outcome measurements.
